# Fuzzy Logic-Based Decision Support for Dairy Cattle Welfare Integrating Different Benchmarks

**DOI:** 10.3390/ani15182729

**Published:** 2025-09-18

**Authors:** Sándor Gáspár, László Pataki, Ákos Barta, Gergő Thalmeiner

**Affiliations:** 1 Department of Investment, Finance and Accounting, Hungarian University of Agriculture and Life Sciences, Páter Károly Str. 1, H-2100 Gödöllő, Hungary; gaspar.sandor@uni-mate.hu (S.G.); thalmeiner.gergo@uni-mate.hu (G.T.); 2 Doctoral School of Management and Business Administration, John von Neumann University, Infopark sétány 1, HU-1117 Budapest, Hungary; 3 Faculty of Social Sciences, Eötvös Lóránd University, Pázmány Péter sétány 1/A, HU-1117 Budapest, Hungary; 4 Doctoral School of Economics and Regional Sciences, Hungarian University of Agriculture and Life Sciences, Páter Károly Str. 1, H-2100 Gödöllő, Hungary; barta.akos@uni-mate.hu

**Keywords:** animal welfare, welfare quality, fuzzy logic, evaluation system, aggregate index

## Abstract

Nowadays, one of the key areas of sustainable agriculture is increasing animal welfare. However, in the absence of generally accepted measurement criteria and systems, measuring animal welfare can be considered a subjective area that makes measuring animal welfare complex. As a result, both increasing welfare and making intervention decisions are not clear for farm management. In our research, we develop a fuzzy logic-based decision support system that is able to handle the subjectivity arising from determining animal welfare. During focus group interviews, experts pointed out that animal welfare assessment systems do not provide adequate support in decision-making. However, the integration of different benchmarks (past, best values and competitors) and the triangular membership functions assigned to them in the assessment significantly supports decision-making. The models were tested with data collected with the Welfare Quality Assessment System of three dairy farms (Austrian, Hungarian, and Slovak). In our result the models show different assessment results; therefore, an aggregate assessment model was created by aggregating the results of the models. The aggregate model incorporates the value judgments and importance of the different models by applying the Choquet integral, thereby providing a more accurate assessment according to the criteria that meet the expectations of decision-makers. Our research shows that animal welfare assessment systems should be based on fuzzy logic and the application of multi-criteria benchmarks until standards reduce the uncertainty in measuring animal welfare levels.

## Simple Summary

Animal welfare is a crucial aspect of sustainability, but its evaluation remains uncertain due to the lack of standardized criteria. This uncertainty leads to subjective assessments and limits the effectiveness of existing systems in supporting decisions. In our study, we developed a fuzzy logic–based decision support model designed to reduce subjectivity in welfare assessments. The model integrates multiple benchmarks, including past performance, best values, and comparisons with competitors, to provide a more consistent evaluation. Tests on three dairy farms in Austria, Hungary, and Slovakia showed that aggregated results, created by combining different models, offer a more accurate and decision-oriented outcome. Our findings demonstrate that applying fuzzy logic and multi-criteria evaluation enhances the reliability of welfare assessments and supports evidence-based improvements in animal care.

## 1. Introduction

One of the narrow cross-sections of sustainable agriculture is animal welfare, which plays a prominent role in regulation and consumer expectations. The adequate welfare status of animals has a direct impact on the environmental sustainability, economic viability and social acceptance of animal husbandry. An adequate level of animal welfare positively influences the health status of animals, reduces the incidence of diseases and thus reduces the use of antibiotics [1]. For the long-term sustainability of agricultural production, it is therefore essential that producers and decision-makers can make decisions based on reliable and objective animal welfare information that approximates reality. However, currently, the assessment of animal welfare on farms has significant subjectivity [2,3,4], which is further complicated by the lack of a generally valid assessment system. The aim of the study is to build a model that is able to deal with the uncertainty arising from subjectivity, thereby facilitating more accurate decision-making. To date, no study has integrated the Welfare Quality^®^ system into a dynamic, multi-standard fuzzy logic framework that simultaneously addresses temporal, normative and contextual uncertainties. The mathematical approach used in this study may contribute to the use of animal welfare indicators to improve the reliability of the indicator system for sustainable agriculture. However, the interpretation of this broader sustainability framework requires an exploration of the current problems and limitations of animal welfare systems [5].

By the beginning of the 21st century, the effects of industrial methods used in intensive animal husbandry on farm animals had caused increasing public concern [6,7]. As a result of this growing concern, a number of animal welfare regulations and standards were developed to ensure the adequate welfare of animals kept in intensive, closed animal husbandry systems. The purpose of animal welfare standards and systems is to ensure the establishment of the appropriate state of health of farm animals, its preservation and the satisfaction of their welfare needs [8]. An animal welfare assessment system must assess animal welfare from the point of view of the animals, monitor changes over time, and identify risk factors [9,10]. However, animal welfare is a controversial topic without a generally accepted definition and consensus [11] and no information is available on the relative importance of animal needs [12,13,14]. Thus, animal welfare can be assessed on the basis of different goals, aspects and mathematical methods, so the different animal welfare assessment systems can be considered as subjective assessment systems [15,16]. Therefore, there is no generally accepted animal welfare assessment system for measuring animal welfare. As a result, supervisory institution can only use heuristic monitoring systems that do not serve as a clear representation of reality.

Consequently, various animal welfare indicators and expert opinions are always subjective [12]. Therefore we can reach a point where assessing animal welfare based on certain moral aspects can lead to the conclusion that meat consumption costs the lives of other living beings and is therefore unethical. Following this line of thought, all animal husbandry can be classified as unethical in the welfare assessment of animal farms, which raises the question of whether animal welfare can be understood at all in this approach [17]. As a result, however, all animal welfare standards lose their relevance, which means that animal farms can only be classified in one final category, the “unethical” category. Following this logic, many interest groups (especially animal rights activists and vegetarians) reject all possible forms of animal husbandry. The assessments from these groups do not fit into a model that assesses welfare. Therefore, in developing the models used in this study, we took into account expert opinions and normative perspectives that accept the assumption that the welfare of farm animals can only be assessed on a continuum scale. This restriction ensures the internal consistency of fuzzy logic-based models. Limiting values of linguistic terms may be determined by the expert judgment of the evaluation systems used, the mathematical methods applied, or context, but since there is no generally accepted method of animal welfare assessment, no generally accepted linguistic terms crisp limit can be defined. Based on all this, refs. [18,19] it can be stated that the assessment of animal welfare can be interpreted as a fuzzy set theory. The research problem of this paper is that animal welfare assessment of livestock farms is a fuzzy set theory problem that results from the subjectivity of different animal welfare assessments and the nonstandard value of threshold limits.

Animal welfare assessment is a fuzzy set theory problem:

(1) the boundaries of animal welfare measurement and its qualifying linguistic expressions are subjective (e.g., unqualified, acceptable, enhanced, excellent)

(2) the input function of fuzzy logic is subjective expert opinion; the subjective animal welfare assessment criteria used in animal welfare assessment are the input functions

(3) are relative in time for the same animal farm

(4) the results of animal welfare assessment systems are relative.

Four conceptual models are presented to illustrate the fuzzy set theory of animal welfare assessment systems. The conceptual models presented in this study were developed based on empirical data collected during focus group interviews with animal welfare experts and then tested on selected animal farms to demonstrate their practical applicability. In our research, we used the Welfare Quality^®^ protocol to collect data on animal welfare levels. It serves as a standardized input framework for collecting and structuring animal welfare data within the models. In order to assess animal welfare, the models include comparisons with other farm, developed benchmark, and the farm’s own previous performance using dynamic reference points. The place occupied on the scale does not depend on the value achieved in the unified indicator system, but on the comparison, developed standard and its own past results of the examined organizations. The maximum and minimum values are given by the system itself and are not predefined. The fourth model is the integration of fuzzy numbers that will be scaled along different benchmarks, thus facilitating the solution of the decision problem arising from the differences in different norms. By using the Welfare Quality^®^ protocol system and various benchmarks together, our aim to demonstrate that the assessment categories used in animal welfare standards do not adequately express the level of animal welfare and its development. By integrating benchmarks into the models, the assessment of the animal welfare level of farms becomes more substantiated, as the animal welfare level and its development trend can be identified in relation to the benchmarks. Decision-makers receive more detailed information on which to make more informed decisions.

Interpretation of the models:

Model 1: We evaluate the past and current animal welfare level of the given farm based on the Welfare Quality^®^. We compare the current achieved result to the past, thereby determining the direction of change compared to its own starting point.

Model 2: We compare the values achieved by the examined farm to the best Welfare Quality^®^ values within the sample.

Model 3: We illustrate the comparison of the Welfare Quality^®^ results of two farms to each other, the degree of difference from each other.

Model 4: We integrate and evaluate the fuzzy numbers determined based on the previous models using the Choquet integral.

## 2. Literature Review

Even in the case of today’s intensive animal husbandry, the aspect that animals are biological organisms that require a proper state of health for their proper functioning should be emphasized. Adherence to animal welfare considerations it is essential to create and maintain good health and to meet the welfare needs of animals [8]. Animal welfare considerations basically focus on meeting the basic needs of animals. These needs include access to food, water, natural light, fresh air, shelter for rest, adequate space for the high level of biological function and, veterinary care in the event of illness. Among the basic needs of animals, relief from pain, anxiety, and suffering should be highlighted [20], and emphasis should be placed on the practice of the ability to behave naturally [21]. The welfare of animals kept in intensive livestock farming depends primarily on the resources and housing provided. The impact of all human actions on animal welfare will only be beneficial if the changes positively affect the internal condition of animals [22,23].

Animal welfare rules should be designed to meet the needs of animals, which are intended to ensure a good life for them. However, determining what an animal’s good life means is no longer clear [24]. In addition to the fact that basic animal welfare needs are provided, it is not yet claimed that an animal lives a good life. This line of reasoning also leads to the suggestion that in the case of intensive animal husbandry, in addition to providing animal welfare criteria and related ethical theories, it is necessary to be aware of what kind of animals do well in the given conditions and what is “good” for these living beings [25,26].

It can be concluded that animal welfare is a term used to define ethical problems and concerns. It assumes a kind of quality of life experienced by animals, especially those kept by humans under intensive animal husbandry conditions [27,28,29]. Animals, having a central nervous system, interact closely with their environment. By expressing intimate feelings and sensitivities, they demonstrate the existence of individuality [24,30,31]. The expression of individuality can be observed among higher mammals in all animal species kept in intensive animal husbandry. According to this view, animals can not only be treated as a factor of production for human consumption; they also have intrinsic value [26,32,33]. Animal welfare is a controversial topic, without an accepted definition, not to mention the consensus on what constitutes “good” or “bad” [11]. Animal welfare is a major issue in animal ethics and, like the “intrinsic value” of animals, it is also an intensely debated subject [26,34,35,36,37,38,39,40,41].

### Description and Evaluation of Animal Welfare Indicators

In order to assess the validity of different welfare standards, their main aspects need to be taken into account. Welfare standards should range from suffering and anxiety (e.g., persistent pain, fear, hunger, and thirst), through high levels of biological function (e.g., absence of disease, injury, malnutrition) to the possibility of positive experiences (e.g., comfort, satisfaction, species-specific behavior) [42]. A welfare standard should evaluate animal welfare, monitor changes over time and identify risk factors [10,14]. Animal welfare is a multidimensional concept that encompasses both physical and mental aspects and should cover all elements of consensus definition [13,43]. Examining livestock farms from an animal welfare perspective is a very complex activity. There is no generally accepted standard animal welfare indicator and no information is available on the relative importance of animals from different welfare aspects [12,44,45,46]. Most animal welfare standards are built around three animal welfare concepts. The first one examines the issue of animal welfare in terms of biological function. In theory, the emphasis will be on meeting biological needs, for example, by examining the availability of adequate quantity and quality of food and access to water, and also by assessing their adequacy and provision of veterinary care. The second concept is based on the ability of animals to feel pain, to suffer. They must be protected from suffering and a positive experience must be ensured. The third concept is to consider the natural way of life. It focuses on the individual nature of animals, in which the practice of natural behavior should be allowed [20,26].

The most widely accepted definition of animal welfare is based on the principle of the five freedoms [47]. It was developed by the UK Farm Animal Welfare Council (FAWC) in 1979 [13] in the Brambell Report [20]. Table 1 illustrates the indicators of the five freedoms and their meaning.

The prevalency of the five freedom paradigm was due to four key factors [39]. Firstly, Mellor (2017) [50] extend to broader dimensions of animal welfare, including subjective experiences, health status, and behavior. Secondly, from a welfare perspective, he highlighted negative experiences (thirst, fear, hunger, pain) and negative states (illnesses, behavioral expressions, injuries). Thirdly, he identified five specific goals for improving well-being. Fourthly, he proposed practical provisions and advice on how to achieve these goals [50]. These so-called normative principles, which are included in the five freedoms, are still only partially complied with by several intensive livestock farms today [51]. With the growing importance of animal ethics, the animal welfare concepts followed by these intensive livestock farms are gaining even more prominence. The five freedom principle is criticized for assessing animal welfare at only one point in time and not taking the duration of adverse effects on welfare into account. As with the five freedom principle, in most animal welfare standard cases it is observed that the detailed welfare status of an animal is recorded within a relatively short period of time or at a specific time which is usually very short compared to its lifespan [41]. Also, a shortcoming of most animal welfare standard is that they do not assess the significance and well-being of the rest of their lives [25].

To determine the degree of animal welfare, the Tiergerechtheitsindex (TGI) system was established on Austrian dairy farms in 1985. This rating system was later known as the Animal Needs Index (ANI) [52]. ANI was one of the first evaluation systems to be applied to laying hens, fattening pigs, and sows on a holistic basis in addition to cattle [18]. Numerous studies have been conducted on the use of ANI through the analyses of different farms to assess the welfare of different farm animals [53,54,55,56]. One of the main criticisms of the ANI system is that it basically evaluates well-being only through different aspects of housing and management, i.e., it is based on resource evaluation [57].

One of the main objectives of the Institute of Animal Welfare is to promote intensive animal husbandry in accordance with welfare standards. In 1988, a group of farmers specialized in the natural behavior of farm animals developed the first animal welfare standards for pig breeding with the help of veterinarians and ethologists. Later, holistically, different standards were developed for several animal species and an index called the Animal Welfare Index (AWI) was created. It is a special index that aggregates welfare considerations into a single index through different perspectives and measurement points [58]. However, the developed system has been widely criticized in the past few years. In a recent study, the authors highlight that AWI as a modern animal welfare indicator system does not necessarily illustrate an adequate level of animal welfare. One of the main reasons for their statement is that the AWI does not seek to express the totality of animal welfare in a uniform way. One of its major shortcomings is that natural behavior and positive welfare indicators (such as the provision of toys or grazing) are not included in the indicator system [58]. According to Mazurek et al. [59] a more thorough assessment of animal welfare should in all cases be supplemented by a farm visit as a qualitative study, thus allowing more specific indicators to be defined for the assessment of animal welfare [59]. However, due to subjectivity, this would also raise a number of problems

The Lombardy Extension Service Index (IBS) was used for the first time in Italy and the methodology only applied to dairy cattle kept in cubicles. This evaluation system is also based on resource-based criteria [18]. Basically, the indicators of the IBS welfare system are defined in a simplified way. The system is mainly criticized for not examining other key welfare indicators, such as broad health, physiological, and ethological areas. IBS has not become globally widespread, but has been regularly used and applied by cattle farms in Lombardy [60].

The World Organization for Animal Health (OIE) takes an approach similar to the principle of the five freedoms. In 2002, the OIE expanded its field of expertise to include animal welfare, given that animal welfare is a key factor in determining the health status of animals [61]. In the definition of an organism, an animal has good animal welfare if it is healthy, comfortable, well-nourished, safe, does not suffer from unpleasant conditions such as pain, fear and anxiety, and is able to express bodily behaviors that are important to the bodily and mental state [62]. OIE contains welfare standards for the slaughter, transport and killing of animals to control diseases. Its standards are basic minimum standards agreed by both developing and developed member countries of OIE [63].

Recently, within the framework of the so-called Welfare Quality Project (WQ), an operational definition of animal welfare has been developed (Table 2), which significantly overlaps with the five freedoms and the OIE definition [64]. Even today, WQ protocols are the best known and most comprehensive method for assessing the overall welfare of different farm animal species (chicken, pig, cattle) [65]. The WQ, unlike other welfare assessment protocols, relies predominantly on animal-based measures. In contrast, resource-based and management-based measures typically reflect risk factors for welfare damage rather than measuring welfare directly [57,66].

Since different animal welfare assessment schemes are based on different assumptions, but all have similar welfare claims, an important research question is how well the results of different systems correlate with each other. Several studies have been conducted to discuss this issue and agree that animal welfare assessment systems based on different assessment criteria give different results when examining the same farm, although they determine approximate welfare claims. The first such comparative study was conducted in 2001, in which Alban et al. [67] compared two resource-based methods (i.e., ANI) with an animal-based method, and the statistical results showed that there was no correlation [67]. In 2019, G. De Rosa et al. [18] compared three systems, including the detailed and most complex WQ, ANI and IBS. The researchers’ results showed that there was no convergent validity between the systems examined. Furthermore, when examining conceptually related dairy cattle welfare assessment systems, there is no closer relationship (i.e., the two resource-based assessments) than between unrelated systems (i.e., the resource-based assessments—Welfare Quality^®^ system) [18]. From these results, it can be concluded that there is no unambiguous and accurate animal welfare assessment system, only systems with approximate accuracy.

Due to technological and methodological developments, animal welfare research has increasingly shifted towards animal-based measurement methods, which increase objectivity in assessment systems. In the case of technological developments, the development of artificial intelligence (AI) and machine learning technologies is the most prominent, which can make it more accurate to monitor the physiological and psychological state of animals. In particular, by recognizing and analyzing animal vocalizations (vocal communication), AI-based voice recognition can represent a major breakthrough in the accuracy of animal welfare assessment systems [68]. Animal vocalizations play a significant role in communication between different animals. Emotionally relevant external events, moods, and hormone concentrations that influence appetitive behavior, thirst, and hunger itself can stimulate a complex central nervous system that regulates feedback to the endocrine and behavioral systems to maintain or restore homeostasis. Thus, different mood or emotional states are accompanied by completely unique behaviors, one of which is vocalization. Thus, vocalizations can be relevant indicators of their welfare level in the case of farm animals [69]. However, by using machine learning algorithms and deep neural networks, it is possible to dynamically analyze the acoustic characteristics of animal vocalizations to assess the welfare level, identifying different unique vocalizations expressing pain, stress or anxiety [68]. AI-based voice recognition in intensive animal husbandry is primarily aimed at determining the welfare level of chicken, pigs and cattle in the literature [70]. Several studies have clearly demonstrated the high reliability of this methodology-technology. For example, an AI-based voice recognition system was able to detect animal sounds indicating pain and stress with very high accuracy in a study conducted on chicken farms [71]. In their study, Dae-Hyun Jung et al. (2021) [72] used AI to investigate cattle welfare. The system they developed was able to infer the physiological and emotional state of cattle from the animals’ unique bellowing sounds with 81.96% accuracy. These studies have shown that vocalization patterns of animals in stress or pain are completely different from those of animals in a normal state, so AI-based monitoring can be of great help to veterinarians and farmers in early recognition of animal needs. Although AI-based voice recognition has many advantages, it also has several limitations. The main challenge is to filter out the various acoustic noises that are present in the farm environment and appear as a confounding factor in the accuracy of the algorithms [72]. Furthermore, due to individual vocalization differences between animals, the models need to be continuously fine-tuned [70]. In the future, further development of AI-based voice recognition will become increasingly intensive, and accuracy may be increased by combining data from multiple sensors, such as thermal cameras and motion sensors [73].

## 3. Materials and Methods

In our research, we are developing an animal welfare assessment system that can express the changes in animal welfare levels by integrating evaluation models suitable for measuring animal welfare. Furthermore, it provides a more accurate assessment and evaluates welfare not only based on a single evaluation standard, but also by applying and integrating different benchmarks.

Our model is based on fuzzy logic, which was proposed by Zadeh (1965) [74] for the development of systems that are much closer to human thinking than previous systems based on binary logic. In the natural and social sciences, many phenomena and processes cannot be evaluated or can only be evaluated inaccurately based on traditional binary logic, and their evaluation using exact methods is almost impossible or does not provide reliable results. Systems developed based on fuzzy logic are able to provide evaluations for complex problems that cannot be answered satisfactorily based on binary logic. The fuzzy logic method of the continuum with an infinite set of values, developed by Zadeh in 1965, determines the classification into a given class based on membership functions. Fuzzy inference systems typically consist of three parts: membership functions, inference rules and defuzzification [74].

In a fuzzy inference system, each input variable has a given set of values. Membership functions represent the set or interval of values of a given linguistic variable [75]. Membership in a set can be determined based on a function f(x), which describes the relationships between the input variables. This operation is called fuzzification [76]. In this domain, variables can be classified into different linguistic categories, for example, “bad”, “acceptable” or “excellent” values can be assumed. The system creates the rules of regular elaboration and we can draw conclusions using each linguistic variable. The results generated by the fuzzy system do not appear as a single specific value, but as a fuzzy set containing the membership degree of a given output variable. For the practical interpretability of the results, a defuzzification step is required, during which the fuzzy output variable is converted into a clear, numerical value. The goal is to characterize the condition with a single value that allows for comparison and objectivity of assessment [77].

Membership functions are constructed to associate each element with a membership degree ranging between 0 and 1. The membership function of a fuzzy subset “A” is commonly defined as μ_A: U → [0, 1], which assigns to each element x ∈ U a membership degree μ_A(x) = μ(x) within A. Unlike classical sets, where an element either belongs or does not belong, in fuzzy sets each element is assigned a degree of membership, typically represented by a real number within the [0, 1] interval. Formally, a fuzzy set A over a universe of discourse X is characterized by a membership function μ_A: X → [0, 1], which maps each element x ∈ X to its corresponding degree of membership in A [78]. The shape of the membership function can vary depending on the application, and common forms include triangular, trapezoidal, and Gaussian functions.

In a fuzzy inference system, statements are based on logical rules, which are called “if-then” rules. Each such rule consists of two main parts: the antecedent (if part) and the consequent (then part). The antecedent (premise) states the conditions under which a given situation can be evaluated. These conditions can be based on one or more input variables. The consequent specifies the result to be obtained if the given conditions are met. Fuzzy set operators express the relationships between fuzzy sets of inputs. The three most commonly used fuzzy set operations are intersection, union, and complement.

The output of fuzzy systems is a fuzzy set that is not a clear numerical value, but a linguistic variable. However, no further operations can be performed on the linguistic variable, so a defuzzification step is required to transform the linguistic variable into a specific, crisp numerical value. There are several methods for defuzzification, including: maximum, average of maxima, center of gravity (COG) or weighted average methods [77,79,80].

### 3.1. Fuzzy Triangle Function

In our research, the determination of the threshold values of the different evaluation categories was carried out based on expert interviews. The most commonly used fuzzy functions for evaluating opinions are the triangle and the trapezoid function. By using the triangle and the trapezoid function, fuzzy sets can be created that have different shapes and properties. However, the triangular function is considered more efficient in some cases in several respects:

- Simpler structure and calculation: The triangular function consists of three points (a, b, c), which form the vertices of the triangle. This makes the calculation of the function values simpler. In the case of the trapezoidal function, respondents must provide additional upper threshold values (a, b, c, d).
- Calculation error: The calculation is simpler due to the fewer parameters. In the case of the trapezoidal function, the possibility of calculation error is greater due to the more parameters (a, b, c, d).
- Easier interpretation: Due to the simpler form, the triangular function is easier to interpret and more intuitive for experts.
- Clearer distinction: The triangular function separates fuzzy sets more clearly from each other by having one vertex. Trapezoidal functions have two “flat” parts, which makes the distinction between fuzzy categories more complex.
- Information needed: If less information is available about the subject under investigation, then the use of the triangular function is justified, as it requires fewer parameters.

In addition to the list of advantages of the triangular function in terms of applicability, the subjectivity of the data collection method and the study problem can be considered an advantage [78]. However, it should be emphasized that the triangular function is not always more suitable than the trapezoidal function. The selection of the appropriate function depends on the analysis problem and the complexity of the calculation. We found the triangular function to be suitable for the aim of analysis.

### 3.2. Measurement Protocol System

The indicators used in the developed conceptual model can be general animal welfare indicators, but in some cases it is necessary for the indicators of this system of criteria to be more specialized in order to assess the specific, unique characteristics of the given animal farm and animal species. Therefore, in our study, we use the protocol system of the Welfare Quality^®^ (WQ) project as a subjective expert assessment system. We chose this protocol system because it serves a comprehensive assessment of animal welfare, measuring welfare from the provision of their basic needs to the behavior of the animals [12], and also because the overlapping definitions and indicators developed by the WQ project are the closest to the consensus definition of animal welfare [13]. It is important to highlight that this evaluation system appears as a subjective expert opinion in the models, on the one hand, and as an independent evaluation benchmark in the aggregate model, on the other hand. In addition to various international standards, most countries have their own animal welfare laws and standards. In addition to international and national standards, there are also private standards, which are mostly developed by consumer groups, livestock groups or scientific societies [63,81,82]. In addition to the WQ protocol, any other animal welfare assessment criteria (international, national, consumer, advocacy, market-oriented, etc.) can be incorporated into our model as subjective expert opinions. The WQ project defined four main principles based on a top-down approach, which were further broken down into twelve independent welfare criteria. Specific indicators and measures were defined to assess the twelve welfare criteria. The number of specific indicators and measures can typically vary subjectively depending on the animal species and the type of farming (Table 2). The overall assessment of animal welfare is based on a bottom-up approach. First, criteria are aggregated from the collected, surveyed data, and then the criterion scores are combined to calculate base scores [44,45,46,83].

The farms are classified into a predefined welfare category based on the scores achieved. The classification into categories is performed using a special mathematical operator, the so-called Choquet integral calculation. The mathematical model allows the livestock farm to be classified into one of four predefined categories [12,84]. In terms of principles, the four predefined categories are calculated in the WQ standard as follows:

- Excellent: If all principles are above 55 and two principles are above 80, the animal welfare level is the highest.
- Enhanced: If all principles are above 20 and two principles are above 55, the animal welfare level is good.
- Acceptable: If all principles are above 10 and three principles are above 20, the animal welfare level exceeds or meets the minimum requirements.
- Not classified: If the animal farms do not meet these minimum requirements, the animal welfare level is unacceptable. [12,44,45,46,85].

### 3.3. Defining Evaluation Benchmarks

We used expert opinions to develop our animal welfare assessment model, which was conducted using the focus group interview method. The purpose of the focus group interview is to assess the experiences and opinions of the participants [86]. The advantage of the focus group interview compared to the individual in-depth interview methodology is expressed in the interaction of the participants, the sharing of experiences and knowledge [87]. The different opinions, positions and the resulting disagreements and agreements provide essential information regarding the subject of the study [88]. The structure of the focus group interview is primarily determined by the research objective and the research plan. In order to explore knowledge, the focus groups should be limited to a time interval of 1–2 h. This time interval supports the active participation of the participants. In terms of the number of participants, 6–12 people are considered the most appropriate [89]. This number ensures the diversity of information and opinions, but this number does not limit the sharing of individual opinions, experiences, and knowledge of the participants. In the case of groups larger than 6–12 people, it is possible that the participants are less active, reluctant to share their thoughts, and therefore valuable information is not revealed [90].

When organizing the focus group interview, we placed special emphasis on the selection of participants. The aim of the selection was to involve participants with appropriate theoretical and practical expertise in the study. A total of eight experts were involved in the interview, who had knowledge from different fields. Experts from three countries (Austria, Slovakia and Hungary) participated in the interview. We consider it important to involve experts of different nationalities, because this way we can assess a broader and international experience. Three of the participants are university researchers who primarily conduct research in the field of animal welfare. Three are veterinarians who have expertise in animal husbandry and the physical assessment of animals. In addition, two are accredited animal welfare experts who can support the research with knowledge from both physical and behavioral aspects. The focus group interview, which involved personal presence, took place twice (21–22 October 2024), and in both cases the study lasted two-free hours with breaks. The main topic of the first interview was the diversity of animal welfare assessment systems, which results in the diversity of animal welfare assessments. Subsequently, the categorization logic of the assessment systems also received special attention. Regarding the categorization, there was a clear consensus that no single animal welfare assessment system adequately expresses the overall change in animal welfare levels. In the second half of the interview, the participants proposed benchmarks which application could provide more detailed information to the management of livestock farms about the change in animal welfare levels. As a result, three benchmark were defined:

- Animal welfare level of the past period: This means comparing the animal welfare value of the livestock farm with the animal welfare value achieved in the previous period. This comparison basis can be used to determine the change in the livestock farm compared to the previous period.
- Animal welfare level of the best farm: The best animal welfare level is determined from the defined sample. This comparison provides feedback on where the animal welfare level of the examined enterprise is compared to the best values.
- Animal welfare level of the competitor: The competitor is selected from the defined sample. The comparison with the selected competitor forms a basis that allows the evaluation of the animal welfare level in comparison with livestock farms with similar characteristics (size, husbandry technology, etc.).

The main topic of the second interview was the analysis of the categorization methods of animal welfare assessment systems. The aspect of subjectivity also arose during the evaluation of the threshold values of the evaluation categories. During the focus group interview, we assessed the experts’ opinions on the definition of the threshold values of the evaluation categories of the benchmarks defined for the first time. For each benchmark, the experts defined five categories (critical, not acceptable, acceptable, good and excellent). They then had to define the values along which the category change occurs in the different benchmark categories. The experts had to provide two values (a, c) for each category to determine the threshold values of the categories belonging to each benchmark:

- Minimum value (a): The percentage value from which the indicator belongs to the given category compared to the reference point.
- Maximum value (c): The percentage value up to which the indicator belongs to the given category compared to the reference point.

The given values ($a_{i}$) and ($c_{i}$) should be considered as „-from”, „-to” values. Within the interval thus determined, the indicator can take any value of the given category. During the study, the interview questions in Appendix A were used. The peak function point ($b_{i}$) was determined by the simple arithmetic mean of the values ($a_{i}$) and ($c_{i}$) determined based on expert opinions. The creation of fuzzy categories was determined based on the points ($a_{i}$,$\;b_{i}$, $c_{i}$) using function (1).

(1) $$\mu \left(\omega_{i}\right)=max\left(min\left(\frac{\omega_{i}-a}{b-a},\frac{c-\omega_{i}}{c-b}\right),0\right)$$

where

- ωi: the current value of the indicator under examination,
- a: the lower value of the triangular function (the value from which the membership of the given category begins to increase),
- b: the peak value of the triangular function (the value at which the degree of belonging to the category is maximum, the value is 1),
- c: the upper value of the triangular function (the value above which membership in the category decreases and then ceases).

Normalizing the values obtained during the focus group interview to a uniform, comparable scale ensures the comparability and consistency of different expert assessments. This is served by the fuzzy min-max membership function we use, which handles gradual transitions between assessment categories and ensures objective comparison of indicators.

### 3.4. Defuzzification

In order to aggregate the fuzzy values of the studied farm and to make the different dimensions comparable, defuzzification is required, so a quantitative value must be created for each model for the final weighting and aggregation of the dimensions. Thus, all values can be interpreted on the same scale, and the expert opinion is part of the final value. The fuzzy numbers that form the basis were created based on the consensus of the experts. The defuzzification method was a category pairing that classified the values of the models into four different evaluation categories. The reason for this method is that the WQ system uses a four-level categorization method; therefore, in order to make the different evaluation models uniformly comparable, a four-category defuzzification division method had to be applied. Accordingly, in the case of the models, where a five level classification was used in accordance with the expert consensus, the two worst categories (critical and not acceptable) were merged, so the final four-level category followed the following structure (Table 3).

The defuzzified values assigned to the four-level category system fall in the interval [0–1] and can then be aggregated. The purpose of aggregation is to express the evaluation dimensions and their synergistic or redundant effects on each other in a single, aggregated indicator. For this purpose, we used the discrete Choquet integral methodology, in which we calculated the defuzzified values of the evaluation categories defined as a function of the different benchmarks. Since we only use four benchmarks, the discrete Choquet integral appears here as a finite sum (Formula (3)); therefore, a description of the background of continuous integral theory is not necessary. The resulting aggregate value illustrates the overall performance of the farm included in the given study along all the benchmarks considered. The structured matching and the use of a uniform category number ensured that the values from the different models fit uniformly into the discrete Choquet integral calculation, so that the aggregation could be implemented in a methodologically consistent and substantively appropriate manner. The reason for using the discrete Choquet integral for aggregation is to address the interdependence and non-additive nature of the dimensions. The different evaluation models used in our research show some overlap in their interpretation and expert judgment, thus having a complementary or redundant effect on the aggregate value. The discrete Choquet integral is the most effective method for modeling and aggregating such interactions, allowing for explicit modeling of synergies and overlaps between dimensions.

In the second part of the second expert focus group interview, we asked the experts to assess the fuzzy measure of the different benchmarks ($\mu_{WQ}$, $\mu_{Past},$ $\mu_{Best}$, $\mu_{Competitor}$). The fuzzy measure (μ) of the benchmarks shows their perceived importance from the perspective of animal welfare assessment. During the interview, we assessed not only the importance of individual dimensions, but also the joint importance of combinations of benchmarks. During the interview, the experts jointly assessed the weight of individual dimensions and dimension combinations on a predefined linguistic evaluation scale. This method has been effectively used in the literature when building fuzzy decision support systems or multi-criteria evaluation systems [91,92]. In our research, we assessed the importance of dimensions and dimension combinations in order to apply the discrete Choquet integral calculation to determine the final animal welfare value judgment. The experts had to judge the importance of the dimensions and dimension combinations on a scale of [0–0.5].

The process of calculating the discrete Choquet integral is carried out along the following steps:

1. The defuzzified input values are sorted in ascending order:

(2) $$x_{\left(1\right)}\leq x_{\left(2\right)}\leq \cdots \leq x_{\left(n\right)}$$

2. The subsets are determined along the defined order.
3. Assignment of the fuzzy measure (*μ*) based on expert consensus.
4. Calculation of the discrete Choquet integral:

(3) $$C_{\mu }\left(x\right)=\sum_{i=1}^{n}\left(x_{\left(i\right)}-x_{\left(i-1\right)})\cdot \mu (A_{\left(i\right)}\right)$$

where $x_{\left(i\right)}$ are the defuzzified values in ascending order; $x_{\left(0\right)}$ is the zero element of the series; $A_{\left(i\right)}$ is the subset of dimensions to which the values $x_{\left(i\right)}$ and greater belong; $\mu (A_{\left(i\right)})$ is the fuzzy measure defined by the focus group for the given subset.

The basic formula (Formula (3)) for the discrete Choquet integral, which is based on the ordering of the defuzzified input values and the differences in the measures of the subsets.

### 3.5. Data Collection

The validation of our model was carried out on data from dairy cattle farms. The data was provided to us by the National Education and Research Foundation (NOKKA). NOKKA has been continuously conducting research and data collection in the field of agriculture in Central and Eastern Europe for decades. The collected data covers the field of animal welfare, and within this, the WQ protocol-based data collection. NOKKA provided the data anonymously and with the avoidance of identification for the validation of the model.

Our research included data from three dairy farms. We analyzed data from dairy farms, located in Hungary, Slovakia, and Austria. The method of husbandry was an important criterion in the selection process, as all farms use a mixture of confined and free-range methods. The aspects included in the WQ protocol were assessed by NOKKA between April-November 2023 and April-November 2024 for each herd in a full WQ assessment [45]. Each of the 1376 dairy cattle included in the study was assessed by professional assessors who are able to evaluate the WQ animal welfare aspects appropriately. At the time of data collection, the total herd size was 1376, Austrian (530), Hungarian (452) and Slovak (394), including not only cattle but also pregnant heifer. In addition to the herd-based assessments, the experts also performed individual-level measurements. The data were therefore collected either at herd or individual level. Individual data were aggregated into herd-level indicators through means and frequencies, supporting their integration into the welfare evaluation system. This was necessary to integrate these data at herd level so that the scores for the 11 WQ criteria could be calculated on a scale from 0 to 100, where 100 points represent the highest possible level of animal welfare for the given WQ criterion. Of the 12 WQ criteria, 11 WQ criteria are included because the WQ protocol does not recommend measurement and calculation for the Thermal comfort criterion for dairy cows [45].

The data were collected either at herd or individual level. Individual data were aggregated into herd-level indicators through means and frequencies, supporting their integration into the welfare evaluation system. This was necessary to integrate these data at herd level so that the scores for the 11 WQ criteria could be calculated on a scale from 0 to 100, where 100 points represent the highest possible level of animal welfare for the given WQ criterion. Of the 12 WQ criteria, 11 WQ criteria are included because the WQ protocol does not recommend measurement and calculation for the Thermal comfort criterion for dairy cows [45].

To ensure that the our developed model applied on a stable and reproducible basis, we next examined the reliability of the expert-based parameterisation used in the fuzzy evaluation. In particular, we tested whether the triangular membership thresholds (a, b, c) are internally consistent within each reference frame (Past, Best, Competitor) and whether experts show concordant judgements when assigning importance ratings for the benchmark weights. This step guards against idiosyncratic rater effects and confirms that the rule set underpinning the fuzzy scoring is acceptable across experts and provides a solid basis for model development. The reliability of the expert-based parameterization was evaluated across the three reference frames. In the time-comparison benchmark (Past), Cronbach’s α = 0.983 indicates excellent internal consistency in the positioning of the triangular thresholds (a, b, c). In the sample-best adjusted benchmark (Best), Cronbach’s α = 0.984 is likewise exceptionally high, and in the designated competitor benchmark (Competitor) Cronbach’s α = 0.989 confirms very tight internal coherence (Table S1). The sample was also evaluated in determining the weight values for the benchmarks. It was assessed with Kendall’s coefficient of concordance (W), which operates on ranks and thus treats the 1–5 scores (Not at all important = 1; Slightly important = 2; Moderately important = 3; Important = 4; Extremely important = 5) as ordinal rather than interval data. The analysis yielded W = 0.699, with χ^2^ = 72.66, *p* < 0.001, indicating very strong consensus in how experts rank-order the benchmarks and benchmarks combinations (Table S2). Taken together, the α values show that experts apply a highly coherent logic when placing (a, b, c) thresholds within each benchmark, while the high Kendall’s W demonstrates a robust, shared ordinal structure in their μ-level evaluations. The sample was also evaluated in determining the weight values for the benchmarks. It was assessed with Kendall’s coefficient of concordance (W), which operates on ranks and thus treats the 1–5 scores (Not at all important = 1; Slightly important = 2; Moderately important = 3; Important = 4; Extremely important = 5) as ordinal rather than interval data. The analysis yielded W = 0.699, with χ^2^ = 72.66, *p* < 0.001, indicating very strong consensus in how experts rank-order the benchmarks and benchmark combinations. In parallel, we computed the Cronbach’s α indicator too. Although the Cronbach’s α value is relatively low (α = 0.421), it still expresses a degree of homogeneity among the indicators (Table S2). It remains applicable for model development and can be further strengthened with an increased sample size.

## 4. Results

In our research, we evaluate the animal welfare level according to the WQ criteria system. By further developing the WQ criteria system based on fuzzy logic, we create a complex evaluation method that makes the assessment of animal welfare more accurate. In order to illustrate our results, we used data from three dairy farms. During the comparative evaluation, we evaluate the animal welfare level of the Hungarian livestock farm depending on the benchmarks.

We do not use the WQ overall score value for the animal welfare assessment, since for the effective operation of fuzzy logic-based systems, it is not sufficient to use the aggregated final value of the WQ system. The application of the overall score alone would raise numerous mathematical and interpretation problems. The aggregated evaluation has complex information and is therefore informative, yet it can hide significant differences between individual dimensions. The reason for this is that the aggregate indicator contains a compensation effect, i.e., a dimension with poor performance can be masked by an outstanding result of another dimension, thereby distorting the assessment of the real state. Furthermore, one of the fundamental application criteria of triangular membership functions is that the input variables move on the same, normalized scale, which represents the interval [0–1] [77]. Direct comparison and aggregation of indicators with different scales and different units of measurement may lead to inaccurate results and numerical instability.

Therefore, it is necessary to group the measured aspects for better transparency. Grouping facilitates better interpretability and comparability. We apply the main principles of Welfare Quality^®^ (2009b) [45], thus we distinguish four groups: Good Feeding (GF), Good Housing (GHo), Good Health (GHe) és Appropriate Behaviour (AB). The total value of the groups created in this way is taken into account:

(4) $$\begin{array}{c} GFi=\sum \left({gf}_{i}^{1}\;{gf}_{i}^{2}{\;gf}_{i}^{3}\ldots \ldots ..{gf}_{i}^{n}\right)\\ GHOi=\sum \left({gho}_{i}^{1}{\;gho}_{i}^{2}{\;gho}_{i}^{3}\ldots \ldots ..{gho}_{i}^{n}\right)\\ GHEi=\sum \left({ghe}_{i}^{1}{\;ghe}_{i}^{2}\;{ghe}_{i}^{3}\ldots \ldots ..{ghe}_{i}^{n}\right)\\ ABi=\sum \left({ab}_{i}^{1}\;{ab}_{i}^{2}\;{ab}_{i}^{3}\ldots \ldots ..{ab}_{i}^{n}\right) \end{array}$$

### 4.1. Evaluation According to WQ

Using the data provided by NOKKA, we determined the animal welfare level of livestock farms according to the WQ measurement method. Table 4 summarizes the results according to the 4 main principles of WQ, the table also contains data from the previous period (t − 1) and the current period (t). When comparing the Hungarian, Slovak and Austrian farms, it is clear that the Austrian farm performed the best, which obtained the “Enhanced” rating in the WQ assessment. According to the assessment rule, the other two farms were classified as “Acceptable” in both examined years. This means that there is no significant difference between the three farms included in the study when applying the assessment rules according to the WQ criteria system. The animal welfare level of the Hungarian farm did not change compared to the previous period, remaining in the “Acceptable” category. However, after a more detailed analysis of the scores belonging to the four main principles, it is clear that, with the exception of the AB principle, it has improved in the other three principles. The Slovak farm did not change category compared to the previous period, it also remained in the “Acceptable” evaluation category. Similar to the Hungarian farm, the Slovak farm also achieved progress in three principles, but a decrease can be observed in the case of the GHe principle. In the case of the Austrian farm, there was a category change compared to the previous period, from the “Acceptable” category to the “Enhanced” evaluation category. After a more detailed analysis of the data, it can be seen that among the evaluation principles, there was progress in the case of GHe, and a decrease in the case of the other three principles. However, this positive change was sufficient for the Austrian farm to be classified in a higher animal welfare evaluation category according to the WQ evaluation criteria system. The results adequately illustrate the subjectivity of the animal welfare level measurement and the WQ system evaluation.

The analysis of the four main principles reveals that the Austrian farm performed best in the GF (55.01) and GHe (56.70) principles, while the Slovak farm had the highest scores in the GHo (51.94) and AB (40.19) principles. However, according to the WQ rules, the Slovak farm did not achieve the “Enhanced” category, which is why its rating remained “Acceptable”. Therefore, based on the results of the WQ assessment, we consider the Slovak farm as the competitor of the Hungarian farm (WG_Competitor_) in our further analysis. Among the farms examined, the best values for the four main principles are considered the best (WQ_Best_) benchmark.

### 4.2. Comparison with the Past Period

The second benchmark is the comparison of the examined farm under study with its own past performance. The aim of this is not to compare absolute values over time, but to measure development interpreted on a normative basis. This means that the values achieved at the time point under study (t) are compared with the results of the previous period (t − 1), but not in absolute terms, but as a ratio to the maximum value within the given period group for each welfare principle.

(5) $$\omega_{i}=\frac{\omega_{i}^{t}}{\omega_{i}^{t-1}}=\frac{\frac{GF_{i}^{t}}{\max GF}\;+\;\frac{GHo_{i}^{t}}{\max GHo}\;+\;\frac{GHe_{i}^{t}}{\max GHe}\;+\;\frac{AB_{i}^{t}}{\max AB}}{\frac{GF_{i}^{t-1}}{\max GF}\;+\;\frac{GHo_{i}^{t-1}}{\max GHo}\;+\;\frac{GHe_{i}^{t-1}}{\max GHe}\;+\;\frac{AB_{i}^{t-1}}{\max AB}}$$

(6) $$\omega_{i}=\frac{\frac{51.53}{55.01}\;+\;\frac{50.95}{52.29}\;+\;\frac{30.80}{56.70}\;+\;\frac{33.38}{40.18}}{\frac{46.95}{57.92}\;+\;\frac{51.65}{53.12}\;+\;\frac{20.30}{43.29}\;+\;\frac{36.02}{44.01}}=1.07$$

Normalization plays a key role, each principle (GF, GHo, GHe, AB) is compared to the best value achieved in the current period. This allows the interpretation of development to be determined not only in relation to one’s own performance, but also in relation to the maximum level achieved in the given period. Through normalization, each dimension contributes proportionally to the development indicator. In addition, this approach also guarantees the stability of the fuzzy logic system, since membership functions can only function properly on a standardized scale. The use of normalized ratios is therefore more accurate not only in its interpretation but also in its computational stability than comparing absolute values over time. During the interviews, experts highlighted that the improvement of absolute indicators over time alone may not be sufficient to determine real development if the reference group has also achieved significant development. This type of feedback is much more useful for decision-makers, as it focuses on one’s own development. Comparing past values in this way also allows us to examine whether the farm has actually moved closer to the best or, on the contrary, has fallen behind them. This norm therefore not only measures the difference between the past and the present, but also takes into account the direction and extent to which this difference has moved the farm’s position relative to the best in the norm group. Thus, the result reflects not only development, but also its quality and relative value.

For the evaluation, we use triangular fuzzy membership functions, which were determined based on expert opinions from the interviews. Based on the expert opinions, we determined the threshold values for the five categories (formula (7)).

(7) $$\begin{array}{c} \mu_{\mathrm{critical}}(\omega_{i})=\max(\min\left(\frac{95\%-\omega_{i}}{10\%},1\right),0)\\ \mu_{\mathrm{not}\;\mathrm{acceptable}}(\omega_{i})=\max(\min\left(\frac{\omega_{i}-85\%}{10\%},\frac{102.5\%-\omega_{i}}{7.5\%}\right),0)\\ \mu_{\mathrm{acceptable}}(\omega_{i})=\max(\min\left(\frac{\omega_{i}-95\%}{7.5\%},\frac{110\%-\omega_{i}}{7.5\%}\right),0)\\ \mu_{\mathrm{good}}(\omega_{i})=\max(\min\left(\frac{\omega_{i}-102.5\%}{7.5\%},\frac{115\%-\omega_{i}}{5\%}\right),0)\\ \mu_{\mathrm{excellent}}(\omega_{i})=\max(\min\left(\frac{\omega_{i}-110\%}{5\%},1\right),0) \end{array}$$

**Figure 1:**
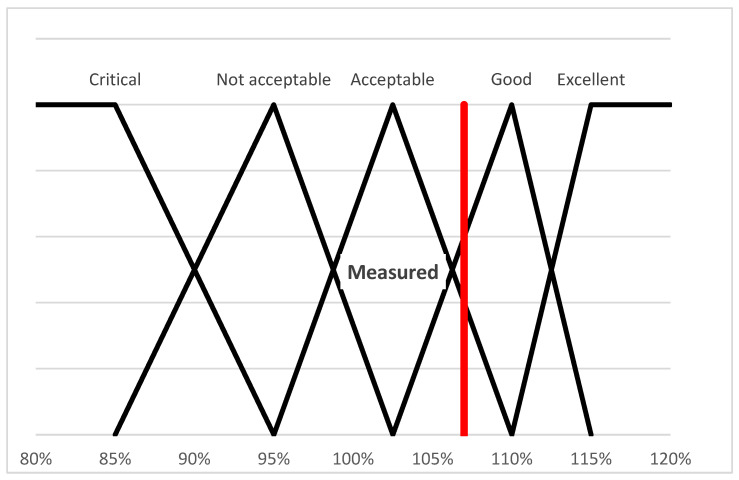
Benchmark of past period value.

The triangular functions belonging to the categories are shown in Figure 1. The value of the Hungarian livestock farm is $\omega_{i}$ = 1.07, which is 107% (marked with the red horizontal line in Figure 1), with which value the animal welfare level of the examined farm was placed in the Good category.

### 4.3. Comparison with the Best Values

The third benchmark is the comparison with the best values within the examined group. This means that for each welfare principle, the reference value is not a theoretical threshold value, but the highest achieved performance within the sample. This approach creates a dynamic assessment, as the benchmark is always interpreted within the given sample. This type of assessment had the greatest support from experts during the focus group interviews. Interviewees highlighted that this approach provides the most useful feedback for development, as it compares one’s own performance not to an externally created benchmark, but to a real, accessible example. For decision-makers, this can lead to more effective decisions, as it directly shows how close they are to the best and in which areas they are lagging behind.

The relative eigenvalue of the farm, which determines their position on the scale, is determined as follows:

(8) $$\omega_{i}=\frac{{GF}_{i}}{\max GF}\;+\;\frac{{GHo}_{i}}{\max GHo}\;+\;\frac{{GHe}_{i}}{\max GHe}\;+\;\frac{{AB}_{i}}{\max AB}\in \left[0,4\right]$$

(9) $$\omega_{i}=\;\frac{51.53}{55.01}\;+\;\frac{50.95}{52.29}\;+\;\frac{30.80}{56.70}\;+\;\frac{33.38}{40.18}=3.29$$

Based on this, the eigenvalue may differ for different samples examined. The principles can take values on a scale of [0–1], so the maximum value of the aggregate eigenvalue is 4.

Based on the opinions of the experts, the intervals belonging to the five categories were determined. Based on the opinions, the triangular functions were also created, which evaluate the relative performance:

(10) $$\begin{array}{c} \mu_{\mathrm{critical}}(\omega_{i})=\max(\min\left(\frac{40\%-\omega_{i}}{20\%},1\right),0)\\ \mu_{\mathrm{not}\;\mathrm{acceptable}}(\omega_{i})=\max(\min\left(\frac{\omega_{i}-20\%}{20\%},\frac{50\%-\omega_{i}}{10\%}\right),0)\\ \mu_{\mathrm{acceptable}}(\omega_{i})=\max(\min\left(\frac{\omega_{i}-40\%}{10\%},\frac{70\%-\omega_{i}}{20\%}\right),0)\\ \mu_{\mathrm{good}}(\omega_{i})=\max(\min\left(\frac{\omega_{i}-50\%}{20\%},\frac{90\%-\omega_{i}}{20\%}\right),0)\\ \mu_{\mathrm{excellent}}(\omega_{i})=\max(\min\left(\frac{\omega_{i}-70\%}{10\%},1\right),0) \end{array}$$

**Figure 2:**
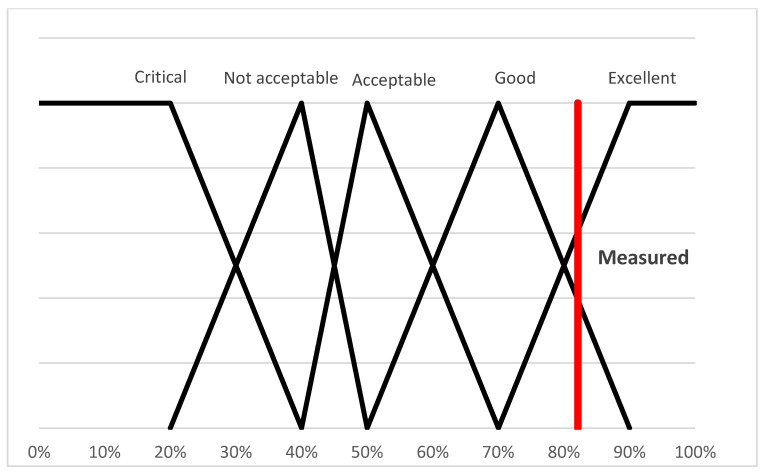
Benchmark of best value.

The value of the examined livestock farm is 3.29, which is expressed in percentage form as follows: $\omega_{i}$/$\omega_{max}\;$= 3.29/4 = 0.8213 so 82.13% (marked with the red horizontal line in Figure 1). It can be seen from the fuzzy membership function (Figure 2) that the value of the examined livestock farm (82.13%) is in the Excellent category.

### 4.4. Comparison with Competitor Performance

In the case of the relative position assessment benchmark, experts unanimously pointed out that models that compare past values or best values are not always able to capture the real differences between livestock farms and facilitate decision-making by assessing the real situation. For example, two farms can both be classified as “Good”, but one just reaches the lower limit of the category, while the other is near the upper limit. According to the focus group participants, a benchmark is therefore needed that can identify the extent to which a given farm performs better or worse than another farm designated as a reference value by comparing two different farms. Based on the experts’ feedback, this is particularly important for farms that want to compare themselves with specific competitors. In contrast, the previous benchmarks (WQ_Past_ and WQ_Best_) focus more on their own position, but are not suitable for illustrating the difference between two competitors.

The experts therefore clearly supported the approach that the comparison should not only be made to the fixed maximum value, but to another selected farm, in such a way that the interpretation is made proportionally within the performance range consisting of the selected farms. Thus, not only the better performance is evaluated, but also the difference between the two farms on a scale from the best to the worst level of animal welfare values in the sample. The farm does not record welfare values on the standard scale, but shows the relative position of the farms examined, to what extent they are better or worse than each other. If the farms are placed on a linear scale, we can see how they are positioned relative to each other in terms of the maximum and minimum values of the group. Furthermore, this normalized difference calculation ensures that the evaluation is dynamic, it always takes into account the composition of the sample. Consequently, the performance of the farm examined can be determined based on this benchmark with the following calculation.

(11) $$\sigma_{i}^{k}=\frac{\omega_{i}-\omega_{k}}{\omega_{max}-\omega_{min}}=\;\frac{\left(\frac{{GF}_{i}}{\max GF}\;+\;\frac{{GHo}_{i}}{\max GHo}\;+\;\frac{{GHe}_{i}}{\max GHe}\;+\;\frac{{AB}_{i}}{\max AB}\right)-\left(\frac{{GF}_{k}}{\max GF}\;+\;\frac{{GHo}_{k}}{\max GHo}\;+\;\frac{{GHe}_{k}}{\max GHe}\;+\;\frac{{AB}_{k}}{\max AB}\right)\;}{\left(\frac{{GF}_{max}}{\max GF}\;+\;\frac{{GHo}_{max}}{\max GHo}\;+\;\frac{{GHe}_{max}}{\max GHe}\;+\;\frac{{AB}_{max}}{\max AB}\right)-\;\left(\frac{{GF}_{min}}{\max GF}\;+\;\frac{{GHo}_{min}}{\max GHo}\;+\;\frac{{GHe}_{min}}{\max GHe}\;+\;\frac{{AB}_{min}}{\max AB}\right)}$$

(12) $$\sigma_{i}^{k}=\frac{3.2851-3.3366}{4-3.0119}=\frac{\left(\frac{51.53}{55.01}\;+\;\frac{50.95}{52.29}\;+\;\frac{30.80}{56.70}\;+\;\frac{33.38}{40.18}\right)-\left(\frac{49.78}{55.01}\;+\;\frac{52.29}{52.29}\;+\;\frac{34.34}{56.70}\;+\;\frac{33.19}{40.18}\right)\;}{\left(\frac{55.01}{55.01}\;+\;\frac{52.29}{52.29}\;+\;\frac{56.70}{56.70}\;+\;\frac{40.18}{40.18}\right)-\;\left(\frac{46.95}{55.01}\;+\;\frac{50.95}{52.29}\;+\;\frac{20.30}{56.70}\;+\;\frac{33.19}{40.18}\right)}=-\;0.05212$$

With this approach, the third evaluation benchmark is fundamentally different from the previous ones. The WQ_past_ benchmark evaluates one’s own development, the comparison with the best values WQ_best_ evaluates the approximation to the best, while the WQ_competitor_ assessment compared to the competitor evaluates a position based on a pairwise comparison depending on the extreme values or reference values in the sample. This benchmark is valuable from a decision-making point of view if it is needed for targeted benchmarking, ranking or competitiveness analysis. The result of the evaluation can take on both negative and positive values. If the result of the analysis is negative, it means that the examined farm performs worse than the animal farm defined as a competitor. If the result is positive, the examined farm has a higher animal welfare value than the animal welfare level of the competitor.

During the focus group interview, the values of the intervals belonging to the categories of this benchmark were also determined (function (13)).

(13) $$\begin{array}{c} \mu_{\mathrm{critical}}(\omega_{i})=\max(\min\left(\frac{(-30\%)-\omega_{i}}{20\%},1\right),0)\\ \mu_{\mathrm{not}\;\mathrm{acceptable}}(\omega_{i})=\max(\min\left(\frac{\omega_{i}-(-50\%)}{20\%},\frac{(-10\%)-\omega_{i}}{20\%}\right),0)\\ \mu_{\mathrm{acceptable}}(\omega_{i})=\max(\min\left(\frac{\omega_{i}-(-30\%)}{20\%},\frac{10\%-\omega_{i}}{20\%}\right),0)\\ \mu_{\mathrm{good}}(\omega_{i})=\max(\min\left(\frac{\omega_{i}-(-10\%)}{20\%},\frac{30\%-\omega_{i}}{20\%}\right),0)\\ \mu_{\mathrm{excellent}}(\omega_{i})=\max(\min\left(\frac{\omega_{i}-10\%}{20\%},1\right),0) \end{array}$$

**Figure 3:**
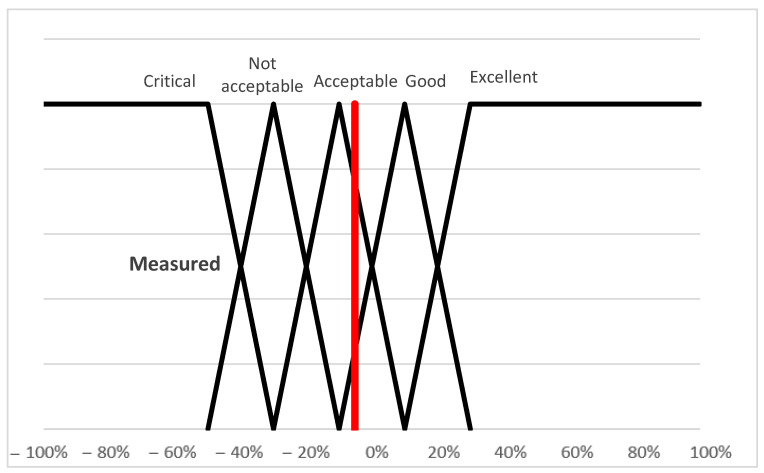
Benchmark of competitor.

As can be seen from Figure 3, the triangular functions are equidistant from each other, which means the intervals of the categories are of equal width.

It can be seen from the fuzzy membership function (Figure 3) that the value of the examined livestock farm $\omega_{i}$ = −0.05212 so −5.212% (marked with the red horizontal line in Figure 3), was placed in the Acceptable category.

### 4.5. Aggregation of the Models

According to the literature, one of the biggest challenges in interpreting and applying different types of animal welfare indicators is how to aggregate different sub-scores from different approaches into a single, interpretable final index. This is particularly important when the evaluation dimensions have complementary logic. That is, they do not replace each other, but rather complement each other. The four benchmark used in this research (WQ, WQ_Past_, WQ_Best_, WQ_Competitor_) each evaluate the animal welfare level along different reference bases, and therefore they cannot be treated with simple, linear weighting. Therefore, the non-additive Choquet integral method is used to aggregate the values. During the evaluation process, the defuzzified values of four different benchmarks were aggregated. The defuzzified values expressed each benchmark in a single numerical value, which carries the expert judgment based on the assigned fuzzy categories. The defuzzification was performed using a matching methodology. This step was essential, as aggregation and final value assessment required accurate values recorded on a uniform scale.

As a first step of the aggregation, the five level evaluation benchmark categories were classified into four-level evaluation categories according to Table 3. Next, we performed defuzzification. During this operation, we assigned specific numerical values to each language category in order to quantitatively transfer the evaluation benchmarks to the aggregation step. The values obtained in this way are in the interval [0–1] and always contain the expert value judgment.

The matching rule used for defuzzification is as follows:

(14) $$f\left(x\right)=\left\{\begin{array}{c} 0.25\;\;\;if\;x=„No\;classified”\\ 0.50\;\;\;if\;x=„Acceptable”\;\;\;\;\;\\ 0.75\;\;\;if\;x=„Enhanced”\;\;\;\;\;\;\;\\ 1.00\;\;\;if\;x=„Excellent”\;\;\;\;\;\;\;\; \end{array}\right.$$

where x = the defuzzified (scaled) value assigned to the given language category.

The defuzzified values of the fuzzy results belonging to the four evaluation benchmarks: the evaluation of the WQ is the “Acceptable” category, to which the paired defuzzified value is 0.50. In the case of the past comparison (WQ_Past_), the evaluation was rated “Good” on the original five category scale, which changed to the “Enhanced” category after the transformation into the four-category system, so the corresponding value became 0.75. Comparison to the best value (WQ_Best_) is the “Excellent” category, to which the value 1.00 was assigned. Comparison to the competitor (WQ_Competitor_) also received the “Acceptable” category, which was paired with a defuzzified value of 0.50. The defuzzified values obtained for the four norms are summarized in Table 5:

The four defuzzified values thus obtained served as input data for the Choquet integral. One of the advantages of the Choquet integral is that it is not additive, it does not assume complete independence between the dimensions. The Choquet integral allows the combination of certain dimensions to have additional information than what they would represent if evaluated separately. With this property, synergies and redundancies can be modeled in practice.

The fuzzy measures (μ) for the different combinations were also determined based on the focus group interviews. The experts were asked to rate each dimension or its combination on a predefined five-point linguistic scale. By aggregating these responses, a fuzzy value was assigned to each combination, which was then modeled with triangular functions and then defuzzified (Appendix B). In this way, the quantitative final fuzzy measures (μ) for each dimension were created.

The following illustrative example is presented for the definition of fuzzy measures (μ). Eight experts rated the importance of a given animal welfare dimension combination (“past comparison” and “position relative to competitors”) on a predefined five-point linguistic scale (“Not at all important”, “Slightly important”, “Moderately important”, “Important”, “Extremely important”). Of the eight experts’ responses, two said it was not at all important, four said it was slightly more important and two indicated it was moderately important. We assigned fuzzy triangular functions to these responses, the parameters of which were defined before the responses based on the predefined scale: not at all important (0.00; 0.05; 0.10), slightly important (0.10; 0.15; 0.20), moderately important (0.20; 0.25; 0.30). The eight fuzzy values were aggregated, then summed as their average in a single triangular function and finally (0.10; 0.15; 0.20) was obtained. The centroid method was used to defuzzify the final fuzzy measure, resulting in a fuzzy measure value of 0.15 for the dimension combination. The fuzzy numbers assigned to the categories are summarized in Table A1 in Appendix B.

The μ values obtained during the analysis of the experts’ opinions for the different dimensions and dimension combinations are shown in Table 6.

The aggregation of the results of WQ protocol and the three models using the Choquet integral was carried out along the following steps:

- We defined fuzzy measures (*μ*) for dimensions and dimension combinations.
- The WQ protocol and the three model results were sorted in ascending order by the defuzzified input value. According to the Choquet integral calculation specified in the WQ protocol [45].
- We calculated the Choquet integral using the fuzzy measures (*μ*) belonging to the ordered dimensions and dimension combinations.

The aggregate welfare score of the farm calculated based on the Choquet integral was 62.50. This numerical value was assigned to a four-level (function (15)) rating system based on a classification system. The reason for this four-level category system is comparability with the WQ, which is one of our research goals. In determining the threshold values for the categories, 100 points were divided into four equal parts. The categories were defined with the following threshold values:

(15) $$s\left(x\right)=\left\{\begin{array}{c} 75.01-100.00\;\;\;\;if\;x=„Excellent”\;\;\;\;\;\;\;\;\;\;\;\;\;\;\;\;\;\;\;\;\;\;\;\;\\ 50.01-75.00\;\;\;\;if\;x=„Enhanced”\;\;\;\;\;\;\;\;\;\;\;\;\;\;\;\;\;\;\;\;\;\;\;\;\;\;\\ 25.01-50.00\;\;\;if\;x=„Acceptable”\;\;\;\;\;\;\;\;\;\;\;\;\;\;\;\;\;\;\;\;\;\;\;\;\\ 0-25.00\;\;\;\;if\;x=„No\;classified”\;\;\;\;\;\;\;\;\;\;\;\;\;\;\;\;\;\;\;\;\;\;\;\;\;\;\; \end{array}\right.$$

The aggregate value of 62.5 is in the “Enhanced” category, which falls in the range of 50–75. This classification means that the animal welfare level of the farm under review has already shown significant improvement, especially compared to past and competitive benchmarks, but still has the potential to improve to reach the highest level (Excellent).

## 5. Discussion

The coexistence of animal welfare assessment systems and the differences in animal welfare criteria raise the question of what level of animal welfare compliance with a given animal welfare assessment system actually suggests. Classification according to a certain animal welfare assessment system does not mean a general level of animal welfare, as classification on the basis of another animal welfare assessment system may result in a different level of animal welfare for the same livestock farm. This is supported by the research of De Rosa et al. (2019) [18], in which the Animal Needs Index (ANI), Lombardy Extension Service Index (IBS), and WQ animal welfare assessment systems were compared. In their study, a quantitative research of thirty-three dairy farms showed that different animal welfare assessment systems that examine similar animal welfare needs yielded different results when testing the same farms. The coefficient of Spearman’s correlation study confirmed the low agreement of the three animal welfare assessment systems. Based on the values of the correlation coefficients, the values of ANI-IBS, IBS-WQ, and ANI-WQ were rs = 0.022, 0.208, and −0.068, and were not significant in either case (*p* = 0.905; *p* = 0.246 and *p* = 0.707). Similarly, in the study of Alban et al. (2001) [67] no significant correlation was found between the resource-based (ANI) system they used and an animal-based evaluation system that was studied in 10 Danish dairy farms. In the study of Johnsen et al. (2001) [93] nine methods for assessing farm animal welfare were described and compared. It was concluded that these methods yielded different results and provided only a good estimate of the average level of animal welfare. Several further studies have reached similar conclusions when comparing different animal welfare assessment systems [94,95,96]. According to Botreau et al. (2008) [83] none of the methods proposed for comprehensive animal welfare assessment are fully suitable for use as a general certification system, not least because most animal welfare assessment systems contain only a few welfare classes. The few welfare classes (acceptable–unacceptable, appropriate–sufficient–not appreciable classes, etc.) do not express the sensitivity assessment of animal welfare sensitively enough. The development of more animal welfare classes proposed by Botreau et al. (2008) [83] for the assessment of more sensitive animal welfare can be integrated in our models, since the number of predefined classes can be increased indefinitely. However, creating too many welfare categories can reduce the accuracy of the models, so an alternative approach is needed, which can be achieved by integrating multiple benchmarks. The model we present integrates benchmarks, which increases the complexity while maintaining the accuracy of the model, allowing for a more accurate assessment of animal welfare levels.

In the selection of subjective expert opinions, the criteria for each area (feeding, housing, behavior, etc.) have a relatively high influence on the calculation of the final score [97,98], hence they greatly influence the classification ability of our models. The ability to classify animal welfare also depends on the definition of threshold limits that are unique to each animal welfare assessment system. The emergence of a fuzzy set theory in the classification and evaluation of animal welfare results from the multiplicity of these animal welfare assessment systems and the differences in the definition of their individual limit values. This is consistent with the study of Cornelissen et al. (2003) [15]; they considered the aspects of public concerns about the welfare of laying hens as a benchmark in their model. In order to develop a general animal welfare assessment system applicable to large-scale animal husbandry, a model is therefore needed that can handle individual thresholds and thus the subjectivity resulting from fuzzy boundaries.

Recent AI-driven techniques in precision livestock farming (PLF) deliver strong task-level performance for event detection under instrumented conditions, for example, pig distress classification from vocalisations and automated audio features, thermal infrared pipelines for claw-lesion/lameness screening and accelerometer-based behaviour/locomotion analytics that enable near real time alerts [99,100,101,102,103]. Reported accuracies in these domains are encouraging, yet typically sensitive to domain shift (acoustics, housing, camera distance/temperature drift) and hinge on continuous, labelled sensor streams and associated IT infrastructure [104,105]. Moreover, farm-level adoption is uneven and often mediated by capex/opex, labour, herd size, and perceived ROI (Return on Investment), while interpretability at the point of use remains variable despite progress in explainable AI [106,107]. These characteristics make AI/ML a powerful solution where dense sensing and robust data pipelines are available, but less attractive where data are sparse, heterogenous, or costly to gather [108]. Our fuzzy model solves a different problem. It is a low-data, multi-benchmark integrator. It takes routine welfare indicators and expert knowledge and turns them into membership functions and clear aggregation rules. This makes three practical differences. First, normative alignment and auditability: thresholds and weights are explicit and can be checked, discussed, and revised. That matters when farms operate under several standards and need a defensible score [12]. Second, data thrift and portability: the method works with indicators many farms already collect, it does not require dense sensing or large labelled datasets. Third, interpretability with tunability: indicator contributions are visible, sensitivity checks (small shifts in cut-points or alternative fuzzy operators) show how stable the score is, and expert-panel statistics can document agreement [15,74,79]. Put simply, AI/ML is best for real-time detection in data-rich barns. The fuzzy model is best for periodic, system-level appraisal when data are scarce or fragmented and multiple standards must be reconciled. On high-tech farms the two approaches are complementary, not competing. Sensor-based risk scores (e.g., lameness, activity) can be used as inputs to the fuzzy aggregator. This preserves transparency while also taking advantage of automated detection [69].

Our goal is to create a system that, by using different benchmarks, provides more detailed information on the level of animal welfare than animal welfare assessment systems and better supports decision-making. In our research, we have developed a model that handles benchmarks defined by experts in animal welfare assessment, using which the animal welfare level can be assessed separately (depending on the benchmark) and in an integrated manner. Benchmarks used in this study enable the expression of changes in the animal welfare level over time and the assessment of performance compared to industry competitors. Thus, the assessment of the animal welfare level includes the subjective animal welfare system (WQ) as an input function and the assessment of own development and the performance compared to industry competitors. In addition to the definition of benchmarks, the definition of category thresholds is also a central element of the model. In our model, we determined the category thresholds based on expert. However, fuzzy logic does not formulate exact values, it indistinctly provides the output values. These ideas are quite far from a satisfactory answer to the question of formalizing inferential processes that involve approximate theories [108].

## 6. Limitation and Future Research

The framework of the study and the validity of its conclusions are fundamentally determined by the fact that we analyzed empirical data from three dairy farms operating with the same technology. The aim of sampling is to ensure that the sample studied is adequately representative of the entire population, as research results are often intended to be extended to the entire sector and to future periods. If the sample is not representative or too small, external validity, the ability to extend the results to other populations, periods and contexts, is compromised [109]. Population validity is particularly sensitive to the sampling method and sample heterogeneity. Larger, more diverse samples provide more reliable and generalizable results, while homogeneous, small-number samples have limited extrapolation value [109]. In addition, due to the lack of statistical power, small samples may miss real effects and random fluctuations may bias the results. Too small sample size can undermine internal and external validity, and too large sample size can make insignificant differences appear statistically significant [110].

The main objective of this study is to provide a theoretical basis for a fuzzy welfare model and demonstrate its practical applicability in the sector. Since the three farms studied do not represent regional, technological or size diversity, the results presented are primarily applicable to such medium-sized mixed-farm dairy farms. The qualitative case study literature calls this approach “analytical generalization”. The emphasis is on creating, testing and in-depth explanations of theoretical models, rather than on population generalization in a statistical sense [111]. The method can therefore contribute to the further development of welfare indicator systems in dairy farming, but in its current form it cannot be considered normative for all production methods.

Future research could continue in two directions. On the one hand, it would be advisable to expand the sample. In the future we could collect data from more regions, different production systems (intensive cage, organic, pasture) and different farm sizes (from family farms to large-scale farms). Increasing the heterogeneity of the sample would improve population validity and strengthen the generalizability of the results. On the other hand, an expanded dataset we could perform prespecified subgroup analyses to assess how the parameters of the fuzzy model vary across husbandry technologies and farm sizes. This would reveal potential structural differences and enable fine-tuning of the model. Finally, the expert-elicitation component currently relies on eight specialists. Enlarging and diversifying this panel would improve the precision and external validity of the elicited membership thresholds and indicator weights, allow formal assessment of inter-expert agreement and consensus and facilitate sensitivity/stability analyses to validate and, where necessary, recalibrate the parameter values proposed herein. Such enhancements would further strengthen the credibility and transferability of the model across production contexts. We emphasize that this study is pilot in nature. The results presented are primarily for demonstration purposes and, in addition to being aware of methodological limitations, serve as a starting point for designing a later, more representative study.

## 7. Conclusions

In this study, we have created an animal welfare assessment model by integrating four different benchmark systems using focus group interviews and the fuzzy logic method. We have shown that animal welfare assessments based on only the WQ simplify reality, since if other evaluation benchmarks are taken into account when evaluating a farm, it may be placed in a different evaluation category. This shows that the assessment of animal welfare is subjective, relative and multidimensional; therefore, it cannot be treated as an objective state tied to a single standard, but the performance of farms must be assessed based on expert opinions and benchmark-dependent interpretations. All this justifies the application of fuzzy logic in this field, which can flexibly handle the difference, uncertainty and subjectivity between value judgments. The approach of the model we have developed can be interpreted primarily as a decision-making tool supporting practical evaluation and comparison, and not as an animal welfare classification system in itself. This is supported by the integrated animal welfare index created in the fourth model, which includes the different results achieved along the benchmarks and the limit values determined by the membership functions. Therefore, by applying this complex indicator, the assessment of the level of animal welfare is presented in a single indicator, aggregating all the benchmark (WQ, WQ_past_, WQ_best_ and WQ_Competitor_) results. This facilitates decision-making and provides more accurate assessments in this complex and ambiguous analysis.

Testing the developed model at extreme values is essential to assess its robustness. Mathematically, this means that the behavior of the model at the threshold values of the input variables is unknown, which causes uncertainty regarding the predictive accuracy of extreme values and cases. This extreme value test can therefore be formulated as a further research proposal. In addition, several benchmarks can be used along the logic applied in the study. The reference benchmarks we chose were preferred by the experts participating in the focus group. In the course of further research, several other benchmarks can be tested, which can refine the accuracy of the model and increase the general validity of the model. The reduction in uncertainty can also be improved by artificial intelligence. New studies highlight that the analysis of animal sounds can improve the accuracy of animal welfare assessments, and this can also affect the models we create. Accuracy primarily results in a decrease in the threshold distance of fuzzy membership functions and, consequently, a decrease in the classification categories, thus increasing objectivity.

The fuzzy logic model presented in this study therefore not only provides a solution to the methodological problems of animal welfare assessments but also establishes a decision support system that can be integrated into indicator systems measuring sustainability. Making animal welfare assessment objectives promotes a more accurate understanding of the environmental, economic and social impacts of agricultural production, as better animal welfare conditions are directly related to a smaller environmental footprint of animal husbandry, more efficient resource use and higher social acceptance. In this way, the presented methodology can contribute to making animal husbandry more sustainable. The fuzzy-based approach is therefore relevant for the development of agricultural and ecological indicators and can be integrated into sustainability monitoring systems.

## Figures and Tables

**Table 1:** The five freedoms and actions.

Freedom	Action
From thirst, hunger and malnutrition	By providing ready access to fresh water and a diet to maintain full health and vigour
From discomfort	By providing an appropriate environment including shelter and a comfortable resting area
From pain, injury, and disease	By prevention or rapid diagnosis and treatment
To express normal behaviour	By providing sufficient space, proper facilities
From fear and distress	By ensuring conditions and treatment which

Source: [48,49].

**Table 2:** Grouping of animal welfare indicators developed by the Welfare Quality^®^ protocol.

Principles	Welfare Criterion	Example of Potential Measures	Example of Measuring Points
Good feeding	Absence of prolonged hunger	BCS (percentage very lean animals)	Percentage of very lean animals
	Absence of prolonged thirst	Access to water	Percentage of groups with sufficient water points
			Percentage of groups with dirty water points
			Percentage of groups with at least two water points
Good housing	Comfort around resting	Frequencies of different lying positions, standing up and lying down behavior	Duration of lying down movement (s)
			Percentage of dirty animals
	Thermal comfort	Panting, shivering	Percentage of panting animals
			Percentage of shivering animals
	Ease of movement	Slipping or falling, possibility of exercise	Space allowance in m^2^/kg
Good health	Absence of injuries	Clinical scoring of integument, carcass damage, lameness	Percentage of lame animals
			Percentage of animals affected with mild and severe alterations
	Absence of disease	Enteric problems, downgrades at slaughter	Number of coughs per animal in 15 min
			Percentage of animals with hampered respiration
			Percentage of animals with diarrhoea
			Percentage of dead animals during a year
	Absence of pain induced by management procedures	Evidence of routine mutilations such as tail docking and dehorning, stunning effectiveness at slaughter	Percentage of tail docked animal
Appropriate behaviour	Expression of social behaviours	Social licking, aggression	Fights and chases per animal and hour
	Expression of other behaviours	Play, abnormal behavior	Number of games per animal
			Percentage of abnormal behavior animal
	Good human–animal relationship	Approach or avoidance tests	Number of animal and human encounters per day
	Absence of general fear	Novel object test	Number of encounters with an unknown person per year
			Number of contacts with unknown objects per year
			Number of relocations per year

Source: [44,45,46].

**Table 3:** Final four-level category.

Expert Category	Aggregation Category
Critical	Not classified
Not Acceptable	
Acceptable	Acceptable
Good	Enhanced
Excellent	Excellent

**Table 4:** Result of the WQ protocol.

Principles	Hungarian (t − 1)	Hungarian (t)	Slovakian (t − 1)	Slovakian (t)	Austrian (t − 1)	Austrian (t)
**GF**	46.95	51.53	49.11	52.45	57.92	55.01
**GHo**	51.65	50.95	50.19	51.94	53.12	51.14
**GHe**	20.30	30.80	43.26	30.55	43.29	56.70
**AB**	36.02	33.38	35.37	40.19	44.01	40.18
**Category**	Acceptable	Acceptable	Acceptable	Acceptable	Acceptable	Enhanced

**Table 5:** Defuzzified values of the fuzzy results.

Evaluation Benchmark Name	Aggregate Evaluation Category	Defuzzified Value
WQ	Acceptable	0.50
WQ_Past_	Enhanced	0.75
WQ_Best_	Excellent	1.00
WQ_Competitor_	Acceptable	0.50

**Table 6:** Fuzzy measures (*μ*) dimensions and dimension combinations.

$\mu_{Competitor}=0.15$	$\mu_{Best}=0.25$		$\mu_{Past}=0.25$		$\mu_{WQ}=0.35$
$\mu_{Competitor,Best}=0.25$		$\mu_{Competitor,Past}=0.25$		$\mu_{Competitor,WQ}=0.35$	
$\mu_{Best,Past}=0.25$		$\mu_{Best,WQ}=0.35$		$\mu_{Past,WQ}=0.25$	
$\mu_{Competitor,Past,Best}=0.35$	$\mu_{Competitor,Best,WQ}=0.45$		$\mu_{Competitor,Past,WQ}=0.45$		$\mu_{Best,Past,WQ}=0.25$

## Data Availability

Dataset available on request from the authors.

## Supplementary Materials

The supplementary materials can be downloaded at: https://www.mdpi.com/article/10.3390/ani15182729/s1, Table S1: Benchmark thresholds dataset, Table S2: Weights dataset.

## Appendix A. Defining Category Thresholds

The following questions aim to define the category boundaries related to the assessment of animal welfare. Please always take into account the benchmark given in the given section. In all cases, please provide the performance as a percentage, where 100% means the same level compared to the benchmark.

### Appendix A.1. Comparison with Previous Year

Instruction: Compare the animal welfare performance to the level achieved on your own farm in the previous year. Example: if conditions are 5% better this year, enter 105%.

Critical ↔ Not acceptable
  - Below what level is the animal welfare condition considered critically low?
  - What is the maximum level that you would still rate as critical?
  - At what percentage does the performance become just not acceptable?

Not acceptable ↔ Acceptable
  - At what level of performance would you say that the performance has reached the acceptable level?
  - Where would you draw the line between the not acceptable and acceptable categories?

Acceptable ↔ Good
  - At what level of improvement would you consider animal welfare to be good?
  - What is the minimum level that you would classify as “good”?
  - Where would you draw the upper limit of the acceptable category?

Good ↔ Excellent
  - At what level of improvement would you say that the animal welfare condition is excellent?
  - Provide a lower and upper limit that separates the “good” and “excellent” categories.

### Appendix A.2. Comparison with the Best in the Group

Instruction: Use the best performing farm in the group as the benchmark (100%).

Critical ↔ Not acceptable
  - Below what percentage level would you consider a farm’s performance to be critical?
  - What is the maximum level that would still classify it as critical?
  - At what level of performance does the animal welfare status become just not acceptable?

Not acceptable ↔ Acceptable
  - At what level of performance does animal welfare performance become acceptable?
  - What is the range that separates the not acceptable and acceptable categories?

Acceptable ↔ Good
  - At what percentage of performance would you consider a farm’s performance to be good?
  - Where would you draw the line between acceptable and good categories?

Good ↔ Excellent
  - Is there a performance that exceeds even the best?
  - If so, at what percentage would you consider it to be excellent?

### Appendix A.3. Comparison to a Competitor Farm

Instructions: Compare your answers to the performance of a known competitor to whom your farm is comparable.

Critical ↔ Not acceptable
  - At what level of backlog would you classify the situation as critical?
  - What is the highest level at which you would classify the performance as just critical?
  - What is the highest level at which the performance would still be classified as critical?

Not acceptable ↔ Acceptable
  - At what percentage level would the performance become acceptable?
  - What is the range that marks the transition between the “not acceptable” and “acceptable” categories?

Acceptable ↔ Good
  - At what percentage level would you say that the farm’s performance is beyond mediocrity?
  - At what percentage level of performance does the “good” level begin compared to the competitor?

Good ↔ Excellent
  - Is there a level that is already significantly better than the competitor’s practice?
  - Where would the performance level that can be classified as “excellent” begin?

## Appendix B. Question List for Defining Fuzzy Measures (*μ*) Values

During the study, we evaluated each farm in three different dimensions—based on four welfare norm systems (WQ, WQ_Past_, WQ_Best_, WQ_Competitor_), as a result of which we determined a defuzzified fuzzy value for each dimension. These four, interpretable scores alone are not sufficient for a comprehensive assessment of animal welfare, as the dimensions have different weights, represent different aspects and partly overlap and partly complement each other in terms of content. Therefore, a coordinated, weighted summary, aggregation of the individual dimensions became necessary. In our research, we used the Choquet integral for aggregation. However, in order to calculate the Choquet integral, it is essential to determine the fuzzy measures (μ values) belonging to the dimension combinations. To establish these weight values, we used the list of questions that we assessed during a focus group interview.

1. One-dimensions (single-element subsets):

- How important do you think the WQ value is in the overall assessment of animal welfare?
- How important do you think the Past value is in the overall assessment of animal welfare?
- How important do you think the Best value is in the overall assessment of animal welfare?
- How important do you think the Competitor value is in the overall assessment of animal welfare?

2. Two-dimensional combinations:

- How important do you think the combination of the Competitor and Best values is?
- How important do you think the combination of the Competitor and Past values is?
- How important do you think the combination of the Competitor and WQ values is?
- How important do you think the combination of the Best and Past values is?
- How important do you think the combination of the Best and WQ values is?
- How important do you think the combination of the Past and WQ values is?

3. Three-dimensional combinations:

- How important do you think the combination of the Competitor, Past and Best values is?
- How important do you think the combination of Competitor, Best and WQ values is?
- How important do you think the combination of Competitor, Past and WQ values is?
- How important do you think the combination of Best, Past and WQ values is?

During the focus group interview, the experts answered a series of questions regarding each dimension and dimension combination, in which they had to evaluate on a five-point scale how important the given dimension is in terms of assessing animal welfare. The fuzzy measures (μ values) were determined in our research based on a pre-structured fuzzy logic system defined by the researchers. As a first step in the process, the researchers developed a five-point evaluation scale that describes the relative importance of the dimensions (not at all important, slightly important, moderately important, important, extremely important). Each category had a predefined fuzzy value range in the interval [0–0.5].

**Table A1:** Fuzzy measures (*μ*) values.

Verbal Category	Fuzzy Value Range (*μ*)	Triangular Fuzzy Number (a, b, c)
Not at all important	0.00–0.10	(0.00, 0.05, 0.10)
Slightly important	0.10–0.20	(0.10, 0.15, 0.20)
Moderately important	0.20–0.30	(0.20, 0.25, 0.30)
Important	0.30–0.40	(0.30, 0.35, 0.40)
Extremely important	0.40–0.50	(0.40, 0.45, 0.50)

After evaluating the expert responses, the researchers chose a specific numerical value from the given range for each dimension by consensus. Each dimension received a final μ weight value, which was used in the calculation of the Choquet integral. The researchers defined each category as a triangular fuzzy number, specifying the minimum, most probable and maximum values. For example: The experts classified the WQ_Competitor_, WQ_Past_ and WQ dimension combination as “very important” in the majority of cases. To this, the researchers assigned a value range between 0.40 and 0.50 and then assigned a μ value of 0.45. From the fuzzy triangular functions belonging to each category, the researchers calculated the defuzzified numerical µ values using the centroid method. Each choice has a well-defined fuzzy weight value. A µ value can now be assigned to each dimension and dimension combination, which can now be directly used for Choquet integral-based aggregation. The determined μ values not only reflect individual opinions, but also take into account the relationships between dimensions and the importance levels perceived by experts.
